# The Articulation of Genomics, Mestizaje, and Indigenous Identities in Chile: A Case Study of the Social Implications of Genomic Research in Light of Current Research Practices

**DOI:** 10.3389/fgene.2022.817318

**Published:** 2022-03-03

**Authors:** Constanza P. Silva, Constanza de la Fuente Castro, Tomás González Zarzar, Maanasa Raghavan, Ayelén Tonko-Huenucoy, Felipe I. Martínez, Nicolás Montalva

**Affiliations:** ^1^ Criminal Justice Research Center, The Pennsylvania State University, University Park, PA, United States; ^2^ Comunidad Autónoma Diaguita Mapochogasta, Santiago, Chile; ^3^ Department of Human Genetics, University of Chicago, Chicago, IL, United States; ^4^ Department of Veterinary and Biomedical Sciences, College of Agricultural Sciences, The Pennsylvania State University, University Park, PA, United States; ^5^ The Huck Institutes of the Life Science, The Pennsylvania State University, University Park, PA, United States; ^6^ Comunidad Kawésqar, Puerto Edén, Chile; ^7^ School of Anthropology, Faculty of Social Sciences, Pontificia Universidad Católica, Santiago, Chile; ^8^ Center for Intercultural and Indigenous Research (CIIR), Santiago, Chile; ^9^ Society and Health Research Center, Universidad Mayor, Santiago, Chile; ^10^ School of Public Health, Universidad Mayor, Santiago, Chile

**Keywords:** genomics, mestizaje, Latin America, Indigenous, identity, research ethics, Chile, ancestry

## 1 Introduction

Genomic research has contributed significantly to our understanding of present-day human biological diversity, health, and disease. However, at the same time, genomic research has historically excluded marginalized groups. In the past decades, the increased access to genomic technologies and data has been paired with efforts to improve sampling diversity by including African descendants and Indigenous peoples. The rationale of these efforts is that disparities in research participation can potentially lead to inequalities in the benefits derived from genomic research (Lee, 2021). Nonetheless, this diversification push has had its pitfalls without clear protocols that improve protection to participants’ DNA data access, data use, and intellectual property against commodification (Fox, 2020). Moreover, Indigenous participation in research continues to be framed in colonial power structures, which are often masked in “reciprocity” and “justice” undermining Indigenous peoples’ sovereignty, self-determination, and governance (Tsosie et al., 2021).

In several Latin American countries, genomic research has advanced two apparently contradictory discourses framed within the *mestizo* rhetoric: *mestizaje*, perceived as genetic homogenization, and Indigenous “purism”, understood as the existence of groups with unmixed genetic ancestry (TallBear, 2013b). These discourses entwine the ideas of genomic ancestry and identity and are perpetuated in academia, either by contributing to the erasure of Indigenous identities under the *Mestizo/Hispanic/Latino* umbrella or by the fetishization of indigeneity by using genetic categories based on a racial logic. The articulation of genetic ancestry with the *mestizo* and Indigenous identities, and their particularities, have been discussed more extensively in the past decades in Brazil, Colombia, and Mexico (Simpson, 2000; Beltrán et al., 2014; Wade et al., 2014b; Kent et al., 2014). It has been proposed that biogeographical ancestries (e.g., European, African, and Amerindian, also called Native American) can evoke different ideas of ancestry, appearance, culture, class, region, and nation, mainly among those outside the genetic research field (Wade et al., 2014b). However, we highlight that the genomic research approaches and interpretations by many experts in Latin America reflect an ongoing global phenomenon in science where peoples’ cultural histories and gene histories are entangled (TallBear, 2013a). This phenomenon of racialized thinking in genomics is embodied in the concept of nation or “genetic citizenships” (Wade et al., 2014b; Wade et al., 2015), which contributes to further stigmatization of historically discriminated populations.

This opinion contributes to this discussion by focusing on two main points. First, we discuss how genomic research opportunistically benefits from the two allegedly contradictory discourses present in the *mestizo* rhetoric; a single *mestizo* national identity based on genetic admixture and Indigenous “purism” based on the articulation of genetic diversity and ethnicity. Second, we situate this problem in the underexplored Chilean sociopolitical context and suggest strategies to improve Indigenous communities’ agency in research settings, contributing to future guidelines on genomic research.

## 2 Genomics and the Latin American Context

### 2.1 Genomic Research and the *Mestizaje* Rhetoric

The invention of Latin America as a geopolitical entity had the aim to fulfill the promise of a “civilization” distanced from the Old World by creating a new cohesive identity (Torres Martínez, 2016). A critical component of this civilizing project was the *mestizaje* (or *mestiçagem* in Portuguese), defined as the admixture of different cultures and racial groups (Wade, 2003). For the Mexican philosopher José Vasconcelos, the *mestizo* represented a “cosmic race” that combined the virtues of Indigenous and Europeans by constituting *“the moral and material basis for the union of all men into a fifth universal race, the fruit of all the previous ones and amelioration of everything past.”* (Vasconcelos and Sánchez, 1966). His ideas reflect the heart of the *mestizaje* as a racial project and building block for the construction of several Latin American national identities; rhetoric that culturally and racially homogenizes populations by erasing the Indigenous by either racial amalgamation or replacement (Tuck and Yang, 2012; Telles and Bailey, 2013). The *mestizaje* rhetoric has mutated in conjunction with the particular sociocultural history of each country, reflected in specific policies and institutions. Furthermore, it has also changed with the development of biotechnologies, including multidimensional ethno-racial concepts such as phenotype classifications, self-identification (Paredes, 2018), and now, DNA ancestry, showing how the implementation of these technologies are far from being neutral (Wade et al., 2014b).

Despite the efforts of scientists to distance themselves from racial categories, the scientific literature continues to use terms such as “DNA ancestry” similarly to racial categories (i.e., continental ancestries), which today are a component of the contemporary *mestizaje* rhetoric. Although the study of genetic variation can provide insights into the relatedness and migration histories of a person’s ancestors, these are not equal to cultural identity or belonging (TallBear, 2013a; Roth et al., 2020). Thus, by positioning DNA as an essentialist marker of shared identity, there is a danger of equating genetic histories with cultural/ethnic identities (Simpson, 2000), a consequence that is permeating the *mestizaje* rhetoric.

In Latin America, the vast majority of human genomic projects have focused on estimating degrees of genetic admixture within nations (Acuña et al., 2000; Wade et al., 2014b; Eyheramendy et al., 2015; Homburger et al., 2015; Adhikari et al., 2016; Berrios, 2016). Such focus aimed at demonstrating that racial categories are useless and that genetic ancestry estimation would be the only deracialized approach to understand genetically mixed societies (Pena, 2000; Wade et al., 2014a; Kent et al., 2015; Mostrador, 2019). Some of these projects not only fall into a genetic fetishism to elucidate historical problematics (TallBear, 2013b), but also reinforce the discourse of a single mixed entity that cannot be differentiated (Kent et al., 2014), endorsing the *mestizo* rhetoric and nationhood (Séguin et al., 2008), yet avoiding any discussion about race (Rodríguez Mega, 2021). In addition, genomic studies have also focused on the genomic articulation of indigeneity or the essentialization of ethnicity into *imagined genetic communities* (Simpson, 2000) or discrete genetic clusters. This has been achieved by differentiating the *mestizo* from the Indigenous or by isolating one or several Indigenous genetic components (Wang et al., 2008; Verdugo et al., 2020).

We observe how genomic research in Latin America opportunistically profits from two apparently contradictory discourses: differentiation and homogeneity, both embedded in the *mestizaje* rhetoric under a scientific rationale. This rationale can achieve truthful results as it supposedly excludes the influence of cultural and social preconceptions (Wade et al., 2014b). However, scientists hold ethical, moral, and political positions that guide their research questions, objectives, approaches, results, and interpretations. The two assumptions ingrained in the *mestizaje* rhetoric are often the starting point of most research efforts in Latin America, such as the case of Chile. On the one hand, the *mestizaje* rhetoric treats genetic admixture as a *continuum*, where the degree of mestizaje can establish, allegedly, that some people are more Indigenous than others. This genetic admixture ladder is articulated with the attribute of indigeneity and, thus, privileges genetic ancestry as a proxy to define Indigenous populations (TallBear, 2013a; Walker et al., 2016). On the other hand, genetic admixture supports the *mestizo* rhetoric of unifying and homogenizing populations under a single national identity (Pena, 2000; Kent et al., 2015; Alpaslan-Roodenberg et al., 2021). Under this logic, the Indigenous/European admixture represented in the *mestizo* signifies the “genetic dissolution” of the original pre-Hispanic Indigenous, foregrounding the idea that either contemporary Indigenous peoples do not exist or are less Indigenous than their ancestors (Tuck and Yang, 2012). This conceptualization can have detrimental consequences in research and sovereignty for Indigenous peoples. For example, a recent publication on ancient DNA suggests that Indigenous heritage is embedded in the *mestizo* national identity of most Latin American countries (Alpaslan-Roodenberg et al., 2021). Therefore, the implementation of similar research standards used in the US for Indigenous engagement and consultation could be counterproductive, and thus Indigenous consultation is not needed (Alpaslan-Roodenberg et al., 2021). However, the authors assume the total integration of Indigenous identities into the *mestizo* national identity, questioning the presence of Indigenous voices and disregarding sociocultural processes in these regions. Moreover, it represents a convenient assumption maintaining the status quo of research practices in Latin America and profiting from the lack of legal protection.

### 2.2 Indigenous Peoples and Genomic Research in Chile

In Chile, around 12.8% of the total population self-identifies as Indigenous. However, none of the 11 Indigenous groups (Mapuche, Aymara, Diaguita, Lickanantay, Quechua, Rapa Nui, Colla, Kawésqar, Chango, Yagán, and Selk’nam) are constitutionally recognized, meaning that collective and territorial rights, sovereignty, and self-determination are not guaranteed by the Chilean State (CIPERChile, 2019). Thus, there is an increased legal vulnerability of Indigenous peoples in Chile, compared to other Latin American countries (Fuentes et al., 2017). This situation is expected to change with the direct participation of Indigenous representatives in the current constituent assembly (Fuentes, 2021).

Chilean genomic research has followed the international trends, prioritizing the genetic characterization of the national *mestizo* population (Ruiz-Linares et al., 2014; Berrios, 2016; Paschetta et al., 2021), but also articulating genetic ancestry and ethnic identity by exploring the “origins” (i.e., ethnogenesis) of these populations (Acuña et al., 2000; Fuentes et al., 2014; Rothhammer et al., 2017; Verdugo et al., 2020). Furthermore, genomic research in Chile has also aimed at identifying informative ancestry markers to characterize specific clusters or ethnic groups (e.g., Mapuche and Aymara), in some cases linking them to diseases (Andia et al., 2008; Bermejo et al., 2017; Díaz-Peña et al., 2020; Jackson et al., 2021; Koenigstein et al., 2021). These concepts of genetic admixture, *mestizo*, or Indigenous DNA are continuously being tossed into the wider society and become part of popular discourses (Simpson, 2000), creating societal narratives about scientifically identifying who are the “real” Indigenous and a way to differentiate who belongs to a specific ethnic group.

Troubling narratives have been derived from genomic research on Indigenous peoples, such as the existence of a “Diaguita DNA” to be studied (ChileGenómico, 2019) and the genetic origin of the Chilean *mestizo* (Berrios, 2016), which serve to essentialize identities based on genetic categories. Genetic groupings can be variable and arbitrary across studies and go as far as labeling physical traits employing ethnonyms, all in a manner that worryingly brings race-based categorization to mind. In addition, studies about the correlation of disease biomarkers with Mapuche ancestry wrongly equate genetic ancestry to ethnicity as the cause of higher gallbladder cancer risk (Bermejo et al., 2017; Jackson et al., 2021). While genetic factors underlie disease susceptibilities, such singular narratives of Indigenous genetic ancestry percentage impacting disease risk drive us away from discussions regarding sociocultural (e.g., diet), socioeconomic, and geographical (rural vs. urban) factors, which could have a broader impact on prevention and health equity policies. Thus, although these research examples preach to be a first step to address racial health disparities, in reality, they evoke race-like categories (Wade et al., 2014b) that further stigmatize Indigenous peoples as genetically different from Chileans/Latinos. However, the bioethics committees at Chilean academic institutions still have to further develop specific protocols to incorporate Indigenous voices in conversations on ethical sampling procedures, informed consent, data privacy, result interpretations, and science communication. Although there is a need to create legal, regulatory, and normative instruments appropriate to the current challenges of genomic research that guarantee Indigenous communities’ participation and protection of their genetic data, these changes will take time. We believe that, in the meantime, the alternative path is to empower Indigenous communities in research settings. This alternative gathers the sociopolitical responsibility for reparation that we, researchers, have in the face of a persistent history of bad practices.

## 3 Strategies for More Ethical and Equitable Research

Often, research has been done on Indigenous peoples, instead of for, with, or by them (Dalton, 2002). Further, in territories where the government does not constitutionally recognize Indigenous peoples, the improvement of research practices in academia needs to start by empowering Indigenous communities in research settings instead of relying on the goodwill of the researchers or expecting academic institutions to make amends. There are great international examples of inclusive research by Indigenous researchers from the United States, Canada, Australia, and Aotearoa on community-based research, mentoring, and mechanisms to empower Indigenous peoples in Western research settings (Claw et al., 2018; Tsosie and Claw, 2020). In order to implement some of Claw et al., 2018 guidelines in the Chilean context, where this discussion is just beginning, we consider a need for a radical shift in how research is conducted. Therefore, we propose to: first, establish long-term partnerships between researchers and Indigenous communities with bidirectional educational purposes (Tsosie and Claw, 2020). This approach will allow the integration of cultural perspectives into research, which has the advantages of creating better-informed, ethical, culturally appropriate, and respectful science (Claw et al., 2018; Begay et al., 2020). Second, academic institutions and researchers should advocate and support educational, mentorship, and training opportunities for Indigenous peoples as researchers. For example, in the US, Canada, Australia, and Aotearoa, researchers have developed the *Summer internship for INdigenous peoples in Genomics (SING)* workshop to discuss the uses, misuses, and limitations of genomics as a tool for Indigenous peoples’ communities. Further, the long-term aim is to propel Indigenous peoples in science research, leadership, and teaching careers at all levels, making genomic research by and for Indigenous peoples. We expect these initial steps to promote a shift in the current research ethos in the region by improving research practices, scientific training, and moving towards community-based collaborative practices that support Indigenous interests and concerns. Many other areas still need to be improved regarding data privacy, data ownership, and research infrastructure within communities. Our suggestions are a first step for paving the path towards more ethical and beneficial research with Indigenous communities.

## 4 Conclusion

At the heart of all the above lies a somewhat urgent need to implement mechanisms in Latin America that can secure positive engagement with genomics while countering misuses of and misinformation from it. We believe that establishing novel collaborative mechanisms between academia and Indigenous groups can introduce researchers to knowledge that recognizes forms of kinship, relatedness, ancestry, and heritage that are not reliant on DNA; forms of knowledge that recognize the different places that people’s histories and gene histories occupy. This collaborative approach is required to debunk myths about genetics and the *mestizo* rhetoric at large.

## Author’s note

Spanish version of this article is available in Supplementary Material S1.
