# Supplementary material for: The Articulation of Genomics, Mestizaje, and Indigenous Identities in Chile: A Case Study of the Social Implications of Genomic Research in Light of Current Research Practices
Source: Front Genet. 2022 Mar 3;13:817318. doi: 10.3389/fgene.2022.817318 (PMC8927767; doi:10.3389/fgene.2022.817318)
Supplement: Supplementary file 1 [file Presentation1.pdf]

La articulación de la genómica, el mestizaje y las identidades Indígenas en Chile: un estudio de caso de las implicancias sociales de la investigación genómica a la luz de las actuales prácticas en investigación

Constanza P. Silva[1,2*], Constanza de la Fuente Castro[3*], Tomás González Zarzar[4,5*], Maanasa Raghavan[3], Ayelén Tonko-Huenucoy[6], Felipe I. Martínez[7,8] & Nicolás Montalva[9,10]

[1]Criminal Justice Research Center, The Pennsylvania State University, University Park, PA, United States.

[2]Comunidad Autónoma Diaguita Mapochogasta, Santiago, Chile.

[3]Department of Human Genetics, University of Chicago, Chicago, IL, United States.

[4]Department of Veterinary and Biomedical Sciences, College of Agricultural Sciences, The Pennsylvania State University,University Park, PA, United States.

[5]The Huck Institutes of the Life Science, The Pennsylvania State University, University Park, PA, United States.

[6]Comunidad Kawésqar, Puerto Edén, Chile.

[7]School of Anthropology, Faculty of Social Sciences, Pontificia Universidad Católica, Santiago, Chile.

[8]Center for Intercultural and Indigenous Research (CIIR), Santiago, Chile.

[9]Society and Health Research Center, Universidad Mayor, Santiago, Chile.

[10]School of Public Health, Universidad Mayor, Santiago, Chile.

Artículo original: "The articulation of genomics, mestizaje, and Indigenous identities in Chile: A case study of the social implications of genomic research in light of current research practices", DOI: 10.3389/fgene.2022.817318

Traducción del original.

1. Introducción

La investigación genómica ha contribuido significativamente a nuestra comprensión de la diversidad biológica, salud y enfermedad de poblaciones actuales. Sin embargo, al mismo tiempo, las investigaciones en este campo han históricamente excluido a grupos minoritarios y/o marginalizados. En las últimas décadas, el aumento en el acceso a tecnologías y datos genómicos se ha visto acompañado de esfuerzos por diversificar la toma de muestras, incluyendo a descendientes africanos y grupos indígenas. Estos esfuerzos son justificados porque las disparidades en participación pueden potencialmente traducirse en desigualdades en los beneficios derivados de la investigación genómica (Lee, 2021). Sin embargo, este esfuerzo por diversificar tiene sus deficiencias ya que no incluye necesariamente protocolos claros que mejoren la protección de la información genética de los participantes, el uso de los datos, y la propiedad intelectual para evitar su mercantilización (Fox, 2020). Más aún, la participación de personas indígena en investigación continúa siendo formulada dentro de dinámicas de poder colonialistas, las cuales usualmente enmascaran su participación bajo argumentos de “reciprocidad” y “justicia”, pero que no hacen más que debilitar la soberanía, autodeterminación, y gobernanza de los pueblos indígenas (Tsosie et al., 2021).

En diversos países de Latinoamérica, la investigación genómica se ha beneficiado de dos discursos aparentemente contradictorios en el contexto de la *retórica mestiza*: el mestizaje, percibido como homogeneización genética; y el “purismo” indígena, entendido como la existencia de grupos con ancestría genética indígena no mezclada (TallBear, 2013a). Estos discursos combinan ideas de ancestría genética e identidad, y son perpetuados en la academia, contribuyendo a ocultar las identidades indígenas bajo la categoría *mestizo/hispano/latino* o mediante la fetichización de lo indígena; discursos que emplean categorías genéticas basadas en lógicas raciales. La articulación de ancestría genética con identidades mestizas e indígenas, así como sus particularidades, ha sido discutida de forma más extensa en las últimas décadas en países como Brasil, Colombia, y México (Beltrán et al., 2014; Kent et al., 2014; Simpson, 2000; Wade et al 2014b). En estos casos, se ha propuesto que categorías de ancestría biogeográficas (ej.: europea, africana, y amerindia o nativa americana) pueden evocar distintas ideas de ancestría, aspecto físico, cultura, clases, región y nación, principalmente entre personas fuera del campo de investigación genética (Wade et al., 2014b). Sin embargo, en esta opinión queremos destacar que este tipo de interpretaciones que articulan ancestría genética con identidades emanan principalmente de las investigaciones genómicas a través de interpretaciones de los propios expertos en Latinoamérica, reflejando un fenómeno global actualmente en las ciencias, en donde la historia cultural de los pueblos y la historia genética se ven entrelazadas (TallBear, 2013a). Este fenómeno de pensamiento

Traducción del original

racializado en genómica queda representados en los conceptos de nación o "ciudadanía genética" (Wade et al., 2015, 2014b), los cuales contribuyen a la estigmatización de poblaciones históricamente discriminadas.

Esta opinión contribuye a esta discusión centrándose en dos puntos principales. Primero, discutiremos cómo la investigación genómica se beneficia oportunistamente de dos discursos supuestamente contradictorios de la retórica mestiza: una identidad nacional única y mestiza basada en el mestizaje y, por otro lado, el "purismo" indígena basado en la articulación de diversidad genética e identidades étnicas. En segundo lugar, situaremos esta discusión en el contexto sociopolítico chileno, donde ha sido escasamente abordado, sugiriendo estrategias para mejorar la participación de comunidades indígenas en investigación, contribuyendo a futuras directrices para la investigación genómica.

2. Genómica y el contexto Latinoamericano

2.1. Investigación genómica y la retórica del mestizaje

La invención de Latinoamérica, como una entidad geopolítica, tuvo el objetivo de cumplir con la promesa de una civilización distanciada del Viejo Mundo, creando así una nueva identidad cohesiva (Torres Martínez, 2016). Un componente crítico en este proyecto civilizatorio fue el mestizaje (o *mestiçagem* en portugués), definido como la mezcla de distintas culturas y grupos raciales (Wade, 2003). Para el filósofo mexicano José Vasconcelos, el mestizo representa una "raza cósmica" que combina las virtudes de indígenas y europeos constituyendo *"las bases materiales y morales para la unión de todos los hombres en una quinta raza universal, fruto de las anteriores y superación de todo lo pasado*" (Vasconcelos y Sánchez, 1966). Sus ideas reflejan la idea central del mestizaje como un proyecto racial y base para la construcción de diversas identidades nacionales latinoamericanas; retórica que cultural y racialmente tiende a homogeneizar a las poblaciones, borrando lo indígena ya sea mediante amalgamiento o reemplazo (Tuck y Yang, 2012; Telles y Bailey, 2013). Esta retórica del mestizaje ha cambiado junto con la historia sociocultural de cada país, lo que se ve reflejado en instituciones y políticas específicas. Más aún, esta retórica también ha mutado en conjunto con el desarrollo de biotecnologías, incluyendo conceptos multidimensionales etno-raciales tales como clasificaciones fenotípicas, auto-identificación (Paredes, 2018), y más recientemente, ancestría genética, dando cuenta cómo la implementación de estas tecnologías están lejos de ser neutrales (Wade et al., 2014b).

Traducción del original

Pese a los esfuerzos de quienes realizan investigación por distanciarse de las categorías raciales, la literatura científica continúa usando términos tales como "ancestría genética" de forma similar a categorías raciales (ej.: ancestrías continentales), que hoy son un componente central en la retórica contemporánea del mestizaje. Aunque el estudio de la diversidad genética puede contribuir a conocer las relaciones ancestro-descendiente e historias migratorias de nuestros antepasados, éstas no son lo mismo que las identidades culturales o pertenencia (TallBear, 2013a; Roth et al., 2020). De esta forma, al posicionar el ADN como un marcador esencialista de identidad compartida, corremos el riesgo de igualar las historias genéticas con identidades culturales o étnicas (Simpson, 2000), un discurso que está permeando la retórica del mestizaje.

En Latinoamérica, la gran mayoría de los proyectos genómicos en humanos se han concentrado en estimar porcentajes de mezcla genética dentro de cada nación (Wade et al., 2014b; Acuña et al., 2000; Berrios, 2016; Eyheramendy et al., 2015; Homburger et al., 2015; Adhikari et al., 2016). Éstas investigaciones han estado orientadas a demostrar que las categorías raciales son inútiles y que la estimación de ancestría genética sería la única aproximación desracializada para entender sociedades genéticamente mestizas (Pena, 2000; Kent et al., 2015; Wade et al., 2014a; Mostrador, 2019). Sin embargo, algunos de estos proyectos no sólo caen en la fetichización de la genética para dar cuenta de problemáticas históricas (TallBear, 2013b), si no que además refuerzan un discurso de una sola entidad mestiza que no puede ser diferenciada (Kent et al., 2014), endosando la *retórica mestiza* como base de la conformación de la nación (Séguin et al., 2008) y evitando cualquier discusión sobre raza (Rodríguez Mega, 2021). Adicionalmente, estudios genómicos se han también enfocado en la articulación genómica de indigenidad o la esencialización de la etnicidad en "comunidades genéticas imaginadas" (Simpson, 2000) o grupos genéticos discretos. Esto se ha logrado mediante la diferenciación entre el mestizo y el indígena o aislando uno o más "componentes genéticos indígenas" (Wang et al., 2008; Verdugo et al., 2020).

Podemos observar cómo la investigación genética en Latinoamérica se aprovecha de dos discursos aparentemente contradictorios: **diferenciación** y **homogeneización**, ambos impregnados en la retórica mestiza bajo una justificación científica. Esta lógica puede, supuestamente, alcanzar resultados veraces ya que se asume libre de la influencia de preconcepciones culturales y políticas (Wade et al., 2014b). No obstante, quienes realizan investigación cargan con valores éticos, morales, y políticos que influencian sus preguntas de investigación, objetivos, aproximaciones metodológicas, resultados, e interpretaciones de los resultados. De esta manera, los dos supuestos arraigados en la retórica mestiza son usualmente el punto de partida de la gran mayoría de las investigaciones en países latinoamericanos tales como Chile. Por un lado, la retórica del mestizaje considera la mezcla genética como un continuo, en donde el grado

Traducción del original

de mestizaje permite establecer, presuntamente, quienes son más indígenas que otros (diferenciación). Este gradiente de mezcla genética se articula con atributos de indigenidad, privilegiando la ancestría genética como una aproximación para definir poblaciones indígenas (TallBear, 2013a; Walker et al., 2016). Por otro lado, el mestizaje genético apoya la retórica mestiza ya que facilita la unificación y homogeneización de las poblaciones bajo una sola identidad nacional (Kent et al., 2015; Pena, 2000; Alpaslan-Roodenberg et al., 2021). Bajo esta lógica, la mezcla indígena/europeo mayoritariamente presente en el mestizo, implica la “disolución genética” del componente indígena precolombino, destacando la idea que los pueblos indígenas contemporáneos no existen o son menos indígenas que sus ancestros (Tuck y Yang, 2012). Esta conceptualización puede tener consecuencias negativas en la investigación y soberanía de los pueblos indígenas. Por ejemplo, una publicación reciente sobre ADN antiguo sugiere que el patrimonio indígena está inserto en la identidad nacional mestiza de la gran mayoría de los países Latinoamericanos (Alpaslan-Roodenberg et al., 2021). Por lo tanto, la implementación de estándares de investigación utilizados en EE.UU. en el contexto de interacción y consulta indígena podrían ser contraproducentes, y por lo tanto, innecesarias (Alpaslan-Roodenberg et al., 2021). Sin embargo, los autores asumen la integración total de las identidades indígenas en la identidad nacional mestiza, cuestionando la presencia de voces indígenas y obviamente los procesos socioculturales en estas regiones. Más aún, representa un supuesto conveniente ya que permite mantener el *status quo* de las prácticas de investigación en Latinoamérica y beneficiarse de la ausencia de protección legal.

2.2. Pueblos Originarios e investigación genómica en Chile.

En Chile, aproximadamente el 12.8% de la población se autoidentifica como indígena. Sin embargo, ninguno de los 11 grupos indígenas (Mapuche, Aymara, Diaguita, Lickanantay, Quechua, Rapa Nui, Colla, Kawésqar, Chango, Yagán, y Selk’nam) son reconocidos constitucionalmente, y por lo tanto sus derechos colectivos y territoriales, soberanía y autodeterminación no están garantizados por el estado chileno (CIPERChile, 2019). Esta situación contribuye al aumento de la vulnerabilidad legal de los Pueblos Originarios en Chile, comparado con otros países de Latinoamérica (Fuentes et al., 2017). Sin embargo, esta situación se espera que cambie con la participación directa de representantes indígenas en la actual Asamblea Constituyente (Fuentes, 2021).

La investigación genómica en Chile ha seguido las tendencias internacionales, priorizando la caracterización genética de la población mestiza a nivel nacional (Berrios, 2016; Ruiz-Linares et al., 2014; Paschetta et al., 2021), pero también articulando la ancestría genética e identidad étnica mediante la investigación de los “orígenes” (i.e.

Traducción del original

etnogénesis) de la población (Verdugo et al., 2020; Fuentes et al., 2014; Rothhammer et al., 2017; Acuña et al., 2020). Adicionalmente, este tipo de investigaciones en Chile se han centrado en identificar marcadores genéticos de ancestría con el objetivo de caracterizar grupos específicos o étnicos (ej.: mapuche y aymara), en algunos casos asociándolos a enfermedades (Andia et al., 2008; Bermejo et al., 2017; Díaz-Peña et al., 2020; Jackson et al., 2021; Koenigstein et al., 2021). Los conceptos de mezcla genética, mestizo, y ADN indígena permean continuamente ámbitos públicos y se vuelven parte del discurso popular (Simpson, 2000), creando narrativas sociales que sustentan la identificación científica de quienes son “realmente” indígenas y que permiten diferenciar quien pertenece a un grupo étnico específico.

Narrativas problemáticas han derivado de la investigación genómica en pueblos originarios, tales como la existencia de un “ADN diaguita” que puede ser estudiado (ChileGenómico, 2019), así como el origen mestizo de los chilenos (Berrios, 2016), y que han servido para esencializar las identidades basadas en categorías genéticas. El agrupamiento genético puede variar y ser arbitrario entre estudios e incluso llegar a etiquetar rasgos físicos empleando etnónimos, de una forma que rememora peligrosamente categorizaciones basadas en raza. Adicionalmente, estudios sobre la correlación de biomarcadores de enfermedades con ancestría mapuche asocia erróneamente ancestría genética a etnicidad como causal un mayor riesgo de cáncer a la vesicular biliar (Jackson et al., 2021; Bermejo et al., 2017). Si bien factores genéticos pueden contribuir a la susceptibilidad a ciertas enfermedades, aquellas narrativas que sólo destacan el impacto del porcentaje de ancestría genética indígena en el riesgo de enfermedades nos alejan de discusiones sobre los factores socioculturales (ej.: dieta), socioeconómicos, y geográficos (rural versus urbano), los cuales podrían tener un mayor impacto global en la prevención de enfermedades y en la formulación de políticas de salud pública equitativas. Aunque estas investigaciones sugieren ser los primeros pasos para abordar las disparidades en salud, en realidad evocan y refuerzan categorías similares a las raciales (Wade et al., 2014b), incrementando la estigmatización de los pueblos originarios como genéticamente diferentes al chileno/latino. Por otro lado, los comités de ética en instituciones académicas chilenas aún no han desarrollado protocolos específicos para incorporar las voces indígenas en conversaciones respecto a los procedimientos de muestreo, consentimiento informado, privacidad de datos, interpretación de resultados, y comunicación científica. Aunque reconocemos que existe la necesidad de crear instrumentos legales, regulatorios, y normativos apropiados a los desafíos actuales de la investigación genómica para garantizar la participación de comunidades indígenas y proteger su información genética, estos cambios tomarán tiempo. Por lo tanto, por ahora creemos que un camino factible es el de empoderar a las comunidades indígenas respecto a las dinámicas y protocolos de las investigaciones genómicas. Esta vía incluye la responsabilidad sociopolítica de reparación que, como

Traducción del original

investigadores, debemos asumir a la luz de la persistente historia de prácticas de investigación poco éticas.

3. Estrategías para una investigación ética y equitativa

A menudo, la investigación se realiza respecto a personas pertenecientes a pueblos originarios, en lugar de para, con, o por dichos pueblos (Dalton, 2002). Más aún, en territorios donde los gobiernos no reconocen constitucionalmente a los distintos pueblos originarios, la mejora en las prácticas de investigación en academia necesita comenzar por el empoderamiento de las comunidades indígenas, en lugar de confiar en la buena voluntad de los investigadores o esperar que instituciones académicas realicen compensaciones. Existen excelentes ejemplos internacionales de investigación inclusiva en EE.UU., Canadá, Australia, y Aotearoa, en donde se han desarrollada aproximaciones de investigación con preguntas formuladas desde las comunidades (*community-based research*), así como mentorías y mecanismos para empoderar a las comunidades indígenas en lógicas de investigación científica occidental (Claw et al., 2018; Tsosie y Claw, 2020). Con el objetivo de implementar algunas de las medidas sugeridas por Claw et al., 2018 en un contexto chileno, donde estas discusiones están apenas iniciando, consideramos que es necesario un cambio radical en la forma en que las investigaciones se realizan. Para ello, proponemos en primer lugar establecer relaciones de largo plazo entre investigadores y comunidades indígenas con propósitos educativos bidireccionales (Tsosie y Claw, 2020). Esta aproximación permitiría la integración de perspectivas culturales diversas en las investigaciones, promoviendo la creación de una ciencia mejor informada, ética, culturalmente apropiada y respetuosa (Begay et al., 2020; Claw et al., 2018). En segundo lugar, instituciones académicas e investigadores deben promover y apoyar oportunidades educacionales, mentorías, y entrenamiento de futuros investigadores indígenas. Por ejemplo, en EE.UU., Canadá, Australia, y Aotearoa, se ha desarrollado el taller "*Summer Internship for INdigenous peoples in Genomics (SING)*" en donde se discuten las aplicaciones, ventajas y limitaciones de la genómica como una herramienta para las comunidades indígenas. Más aún, el objetivo a largo plazo de SING es apoyar a personas indígenas en investigación científica, liderazgo, y carreras académicas en diversos niveles, promoviendo la investigación genómica realizada por investigadores indígenas y pensando en los intereses de las comunidades. Esperamos que estos pasos iniciales promuevan un cambio en el *ethos* actual de investigación en la región, mejorando las prácticas científicas y aprendizaje, avanzando hacia prácticas colaborativas basadas en comunidades que apoyen los intereses y preocupaciones de las comunidades indígenas. Sabemos que otras áreas también requieren mejorar, tales como la privacidad y propiedad de los datos, y la infraestructura para la investigación dentro de las comunidades. Sin embargo, nuestras sugerencias podrían ser un primer

Traducción del original

paso para preparar el camino hacia una investigación más ética y beneficiosa para las comunidades indígenas.

4. Conclusiones

En el centro de todas estas reflexiones se encuentra la urgente necesidad de implementar mecanismos en Latinoamérica que puedan asegurar relaciones positivas en investigación genómica, contrarrestando a la vez su mal uso y desinformación. Creemos que el establecimiento de nuevos mecanismos de colaboración entre la academia y grupos indígenas puede introducir a los investigadores a conocimientos que reconocen formas de parentesco, relaciones, ancestría, y herencia que no dependen del ADN. Conocimientos que reconocen los distintos espacios que ocupan la historia de las personas y las historias de los genes. Esta aproximación colaborativa es un requisito para desmitificar la genética y la retórica mestiza a un nivel más amplio.

Financiamiento

CIIR/Fondap 15110006

Agradecimientos

University of Chicago Provost's Global Faculty Awards: Latin America.

Traducción del original
